# The Four Wives’ Portraits

**Published:** 1878-09

**Authors:** 


### WHICH AM I? OR, THE FOUR WIVES' PORTRAITS.

(SELECTED.)

IN Horsley Down churchyard, England, is the following inscription:

FIRST PORTRAIT.

Here lie the bodies of
Thomas Bond and Mary his wife.
She was temperate, chaste and charitable.
But
She was proud, peevish and passionate.
She was an affectionate wife, and a tender
mother.
But
Her husband and child whom she loved, seldom
saw her countenance without a
disgusting frown,
Whilst she received visitors whom she despised
with an endearing smile.
Her behavior was discreet towards strangers,
But
Imprudent in her family.
Abroad her conduct was influenced by good
breeding,
But
At home by ill temper.

She was a professed enemy to flattery, anᴈ was
seldom known to praise or commend;
But
The talents in which she principally excelled
Were difference of opinion and discovering
flaws and
Imperfections.
She was an admirable economist,
And, without prodigality,
Dispensed plenty to every person in her family,
But
Would sacrifice their eyes to a farthing candle.
She sometimes made her husband
Happy with her good qualities,
But
Much more frequently miserable with her
Many failings.
Inasmuch that in thirty years cohabitation,
He often lamented that,
Maugre all her virtues,
He had not on the whole enjoyed two years
Of matrimonial comfort.
At length
Finding she had lost the affection of her hus-
band, as well as the regard of her neigh-
bors, family disputes having been
divulged by servants,
She died of vexation, July 20, 1768,
Aged 48 years.
Her worn-out husband survived her four months
and two days, and departed this life
November 28, 1768,
In the 54th year of his age.
William Bond, brother to the deceased,
Erected this stone as a
Weekly monitor to the wives of this parish,
That they may avoid the infamy of having
Their memories handed down to posterity
With a patchwork character.

SECOND PORTRAIT.

She is no true wife who sustains not her husband in the day of calamity; who is not, when the world's great frown makes the heart chill with anguish, his guardian angel, growing brighter and more beautiful as misfortunes crowd around his path. Then is the time for a trial of her gentleness—then is the time for testing whether the sweetness of her temper beams only with a transient light, or like the steady glory of the morning star, shines as brightly under the clouds. Has she smiles just as charming? Does she say, "Affliction cannot touch our purity, and should not quench our love?" Does she try, by happy little inventions, to lift from his sensitive spirit the burden of thought?

There are wives—no! there are beings who, when dark hours come, fall to repining and upbraiding—thus adding to outside anxiety the harrowing scenes of domestic strife—as if the blame in the world would make one hair white or black, or change the decree gone forth. Such know not that our darkness is heaven's light—our trials are but steps in a golden ladder, by which, if we rightly ascend, we may at last gain that eternal light, and bathe forever in its fulness and beauty.

### THIRD PORTRAIT.

"*Is that all?*" and the gentle face of the wife beamed with joy. Her husband had been on the verge of distraction—all his earthly possessions were gone, and he feared the result of her knowledge, she had been so tenderly cared for all her life! But, says Irving's beautiful story, "a friend advised him to give not sleep to his eyes, nor slumber to his eyelids, until he had unfolded to her his hapless case."

And that was her answer, with the smile of an angel—"*Is that all?* I feared by your sadness it was worse. Let these things be taken—all this splendor, let it go! I care not for it —I only care for my husband's love and confidence. You shall forget in my affection that you ever were in prosperity—only still love me, and I will aid you to bear these little reverses with cheerfulness."

Still love her! a man must reverence, aye, and liken her to the very angels, for such a woman is a living revelation of heaven.

### FOURTH PORTRAIT.

As painful a scene met my view in the cars from Philadelphia to New York, as I had ever seen in my journeys. A *lady* and her husband came into the cars at the former place, and were seated near us—very respectable in appearance, and the lady, in particular, uncommonly interesting. After a little while I

noticed a strange manner in the gentleman, which seemed to indicate he was *not in favor* of the Maine Liquor Law. At every place the cars stopped he evidently replenished the vacuum in his throat by a new drink, until he could not sit without help in his seat. He then rose hastily and went and opened the car door, and seated himself in it, with his feet hanging outside. His wife was much distressed, and tried to prevail upon him to come in, and he gave her a push which almost sent her to the floor. Two gents rose, and, with the aid of the conductor, he was helped in and placed in a reclining position on one of the seats beneath a window. He soon apparently fell asleep—and it was enough to break one's heart to see the attentions that that devoted wife lavished upon her senseless husband. She covered him up with her shawl, to keep the dust from making him uncomfortable; if his hands fell in an unpleasant position, she gently replaced them, and perhaps bedewed them with a tear. Before arriving in New York she seemed anxious to have him wake, and asked one of the gents to "please wake him, as it was a strange city, and she did not know what to do." Two or three roused him a little, and then she went to him with a sweet smile, and says: "We have got almost to New York, and I am glad, *you are so tired;*" and he *struck her in the face.* She had the sympathy of all in the car. I know, for there was many a moist eye among the ladies, and many a bitter look on manhood's cheek. Arrived in New York, he would not leave the cars till he was ordered by the conductor; and her attentions in crossing the ferry were as assiduous as ever, and met with pushes and blows from her brutal husband. The last I saw of her she was in the station-house on the New York side, *begging* him to go and see to their baggage, and he answered her she was a fool —to mind her own business, &c. My travelling companion remarked, "That is womanly love, and when he *speaks kindly to her again, she will forget it all.*"

